# Direct Optical Lithography Enabled Multispectral Colloidal Quantum-Dot Imagers from Ultraviolet to Short-Wave Infrared

**DOI:** 10.1021/acsnano.2c07586

**Published:** 2022-11-08

**Authors:** Shuo Zhang, Cheng Bi, Yimei Tan, Yuning Luo, Yanfei Liu, Jie Cao, Menglu Chen, Qun Hao, Xin Tang

**Affiliations:** † School of Optics and Photonics, Beijing Institute of Technology, Beijing100081, People’s Republic of China; ‡ Zhongxinrecheng Science and Technology Co., Ltd., Beijing101102, People’s Republic of China; § Beijing Key Laboratory for Precision Optoelectronic Measurement Instrument and Technology, Beijing100081, People’s Republic of China; ∥ Yangtze Delta Region Academy of Beijing Institute of Technology, Jiaxing314019, People’s Republic of China

**Keywords:** colloidal quantum dots, UV−infrared, optical lithography, dual-band imager, focal plane array

## Abstract

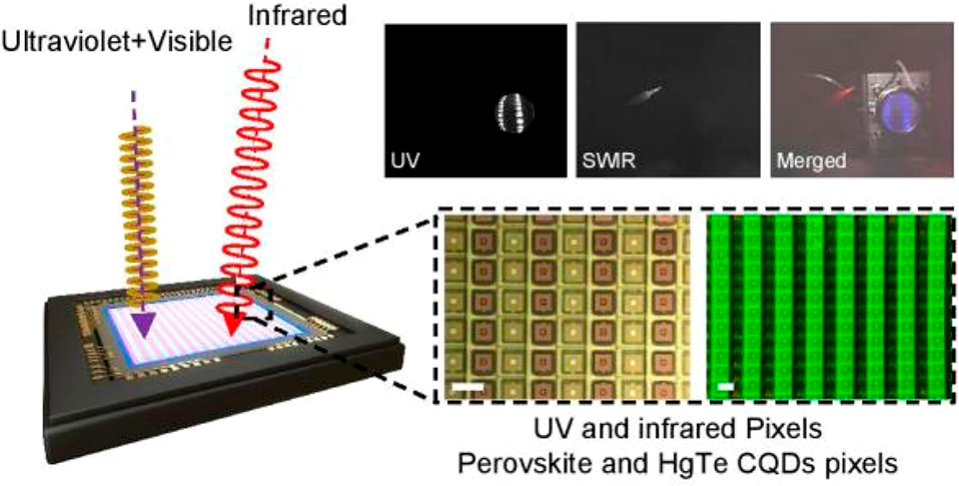

Complementary metal oxide semiconductor (CMOS) silicon sensors play a central role in optoelectronics with widespread applications from small cell phone cameras to large-format imagers for remote sensing. Despite numerous advantages, their sensing ranges are limited within the visible (0.4–0.7 μm) and near-infrared (0.8–1.1 μm) range , defined by their energy gaps (1.1 eV). However, below or above that spectral range, ultraviolet (UV) and short-wave infrared (SWIR) have been demonstrated in numerous applications such as fingerprint identification, night vision, and composition analysis. In this work, we demonstrate the implementation of multispectral broad-band CMOS-compatible imagers with UV-enhanced visible pixels and SWIR pixels by layer-by-layer direct optical lithography of colloidal quantum dots (CQDs). High-resolution single-color images and merged multispectral images were obtained by using one imager. The photoresponse nonuniformity (PRNU) is below 5% with a 0% dead pixel rate and room-temperature responsivities of 0.25 A/W at 300 nm, 0.4 A/W at 750 nm, and 0.25 A/W at 2.0 μm.

## 1 Introduction

Numerous studies have aimed to extend the spectral sensing range of the standard silicon-based CMOS imagers. To meet the huge demands inaccessible by silicon, GaN, InSb, HgCdTe, and type II superlattices have been explored and successfully used for photon detectors.^1^ However, such optical semiconductors are not compatible with silicon CMOS processing and require flip-bonding to achieve an electrical connection between sensing materials and readout chips, leading to high cost and low fabrication yields. Extending silicon’s sensing ranges with CMOS-compatible materials and fabrication flows faces great technological challenges.

With the advancements in low-dimensional semiconductors, including two-dimensional (2D) films, nanowires, and quantum dots,^2^ the integration of silicon with low-dimensional semiconductors provides a promising route toward broad-band hybrid-dimensional photodetectors with extended sensing ranges beyond that of silicon. By epitaxial growth or direct solution processes, WSe_2_ and BA_2_PbBr_4_ have been utilized to extend the sensing ranges of a silicon CMOS imager to the near-infrared and ultraviolet regions, respectively. The WSe_2_/Bi_2_Te_3_ heterojunction-based self-powered photodetectors can extend the detection range from the visible to the near-infrared (0.375–1.550 μm).^3^ A wafer-sized 2D BA_2_PbBr_4_ perovskite single crystal grown by a gas–liquid interface crystalline method realized sensitive UV response with a detectivity up to 10^12^ jones.^4^ However, due to the large discrepancy in photon energy, it is still challenging to combine the detection capabilities of ultraviolet (UV, 3–6 eV) and short-wave infrared (SWIR, 0.5–0.8 eV) into the same imager.

Benefiting from their solution processability, CMOS-compatible integration or monolithic integration of colloidal quantum dots (CQDs) with silicon readout integrated circuits (ROICs) has been carried out, leading to low-cost and large-area hybridization. CQDs-sensitized organic photodiodes,^5^ CQDs-graphene phototransistors,^6^ and CQDs photoconductive imagers^7,8^ have all been demonstrated from the near-infrared to midwave infrared. However, prior research on CQDs optoelectronics have mainly focused on single-color applications. The integration of CQDs with different band gaps onto the same ROICs remains challenging. Despite the fact that several approaches such as inkjet printing,^9^ contact printing,^10^ transfer printing,^10,11^ and electrohydrodynamic jet print (E-jet printing)^12^ have recently been proposed to achieve high-resolution spatial control of deposited CQDs, the yield of those methods is limited and cannot be extended to wafer-scale fabrication.

To address such challenges, a CMOS-compatible multispectral imager with spectral response from the ultraviolet and visible to SWIR has been developed with CQDs. The two CQD channels were fabricated by direct photolithography through sequential spin-coatings of CQDs followed by UV exposure, in which ethane-1,2-diyl bis(4-azido-2,3,5,6-tetrafluorobenzoate) was utilized as a UV-activated ligand to cure the CQD films, which enables facile discrimination of UV light, visible light, and SWIR. The multispectral colloidal quantum-dot imager exhibits high responsivity in the UV and SWIR (cutoff wavelength 2.5 μm).

## 2 Device Design and Operation Principle

**Figure 1:**
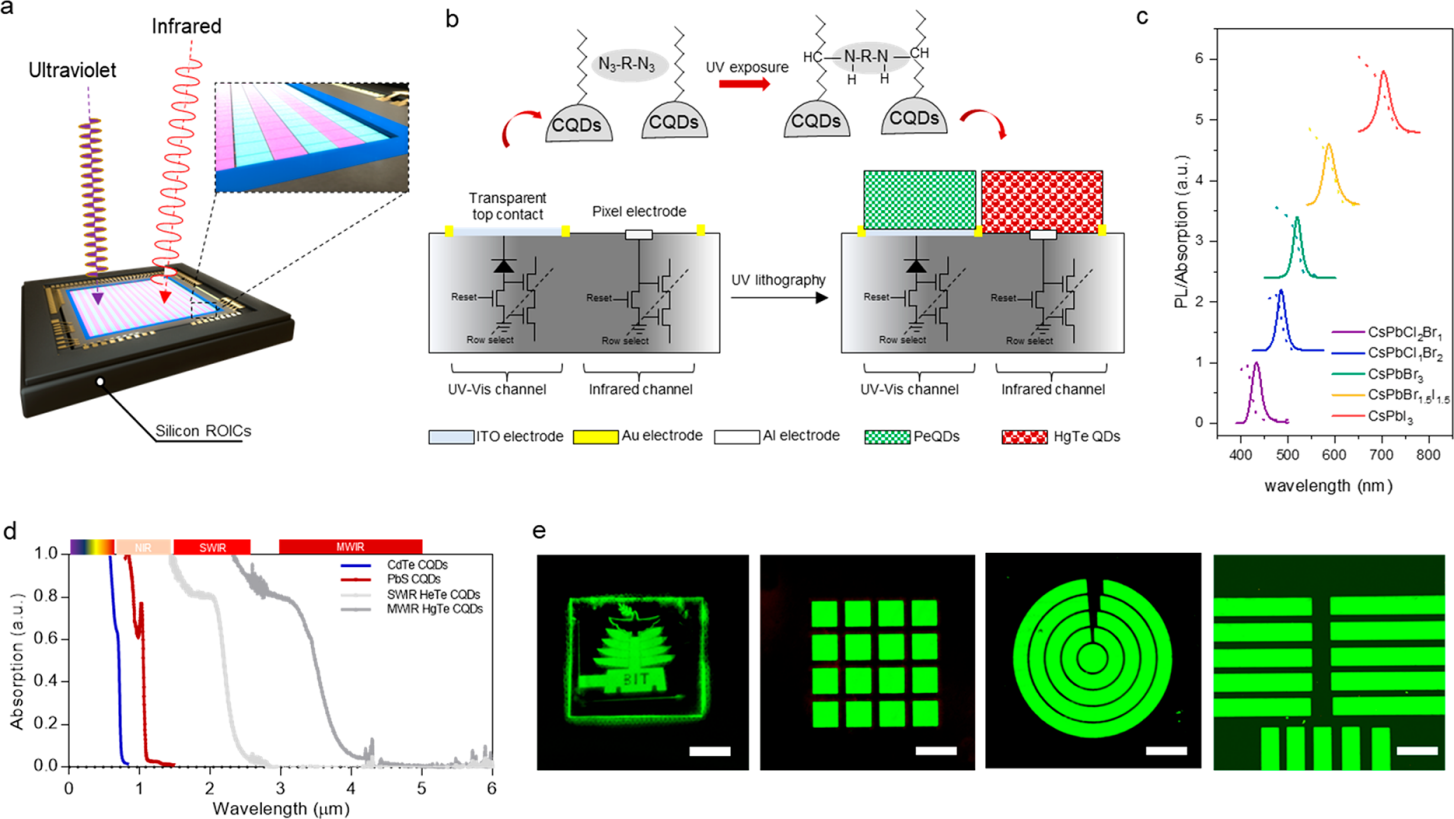
Design and working principle of broad-band multispectral imagers. (a) Device configuration of the multispectral imagers. (b) Schematic illustration of the ligand cross-linking process and the configuration of a UV-enhanced visible pixel and SWIR pixel. (c) Photoluminescence (PL) and absorption spectra of perovskite CsPbX_3_ (X = Br, Cl, I) CQDs. (d) Absorption spectra of SWIR and MWIR HgTe CQDs. (e) Optical images of CQD patterns. The scale bars of the images are 5 mm, 100 μm, 100 μm, and 100 μm, respectively.

The device configuration is illustrated in Figure 1a. On top of silicon ROICs, two separate channels were laterally arranged to detect UV and SWIR light, respectively. As illustrated in Figure 1b, the customized ROIC chip consists of two types of pixels. One is a visible pixel with a transparent top contact, on top of which perovskite quantum dots (PeQDs) were added as downconverters to enhance the UV response. CQD-based downconversion detectors have been widely used to boost the detection efficiency of silicon detectors by converting UV photons to visible photons. Combined with perovskite nanocrystals embedded in composite films, the external quantum efficiency at 295 nm of the silicon photodetector can be greatly improved from 3.3 to 19.9%, which effectively extends the sensing range of the Si detector in the UV.^13^ The other channel is a readout pixel with in-pixel amplification arranged in direct injection mode. For the SWIR CQDs channels, a photoconductive configuration was adopted, and each pixel consists of a center pixel and a surrounding ground ring, between which the bias voltage was applied (Figure S3). The UV-enhanced visible channel provides eye-like vision and gives the basic understanding of a captured scene with extra information such as fingerprint identification,^14^ while SWIR images could cover a 1–2.5 μm region, where infrared signatures of objects can be obtained to determine the composition.

In our experiments, the two CQD channels were fabricated on ROICs by direct photolithography through sequential spin-coatings of CQDs followed by UV exposure, in which ethane-1,2-diyl bis(4-azido-2,3,5,6-tetrafluorobenzoate) is utilized as a UV-activated ligand to cure the CQD films. The chemical structure of ethane-1,2-diyl bis(4-azido-2,3,5,6-tetrafluorobenzoate) contains two fluorinated perfluorophenyl azide groups at both ends of the molecule. The chemical structure, proton nuclear magnetic resonance (^1^H NMR), and fluorine nuclear magnetic resonance (^19^F NMR) of the UV-activated ligands are shown in Figures S1–S3. With two azide end groups, the light-driven cross-linker can interlock the ligands of neighboring QDs under UV (254 nm) exposure. A high resolution and wafer-scale integration can be achieved.

The absorption spectra of PeQDs and HgTe CQDs are shown in Figure 1c,d. CsPbX_3_ (X = Br, Cl, I) CQDs with various emission wavelengths were synthesized by tuning their composition. The emission wavelength can be tuned from 430 to 700 nm. In our experiments, green PeQDs with an emission wavelength at 550 nm were selected based on the photoluminescence quantum efficiency of PeQDs and spectral external quantum efficiency of silicon photodiodes. For infrared detection, CdTe, PbS, and HgTe CQDs are all possible candidates. Among them, HgTe CQDs have so far demonstrated the highest spectral tunability into the midwave infrared. HgTe CQD midwave infrared (MWIR) photodetectors with background-limited photodetection (BLIP) performance,^15,16^ room-temperature SWIR photodetectors,^17,18^ and dual-band infrared detectors^19,20^ have been constructed and showed high-performance imaging. The surface ligands of the as-synthesized HgTe and perovskite CQDs are oleylamine and dodecanethiol, which provide abundant C–H bonds for the UV-activated ligands to interlock with. As shown in Figure 1e, the high-resolution direct pattern of CQDs can be achieved with a minimum feature size down to 2 μm.

**Figure 2:**
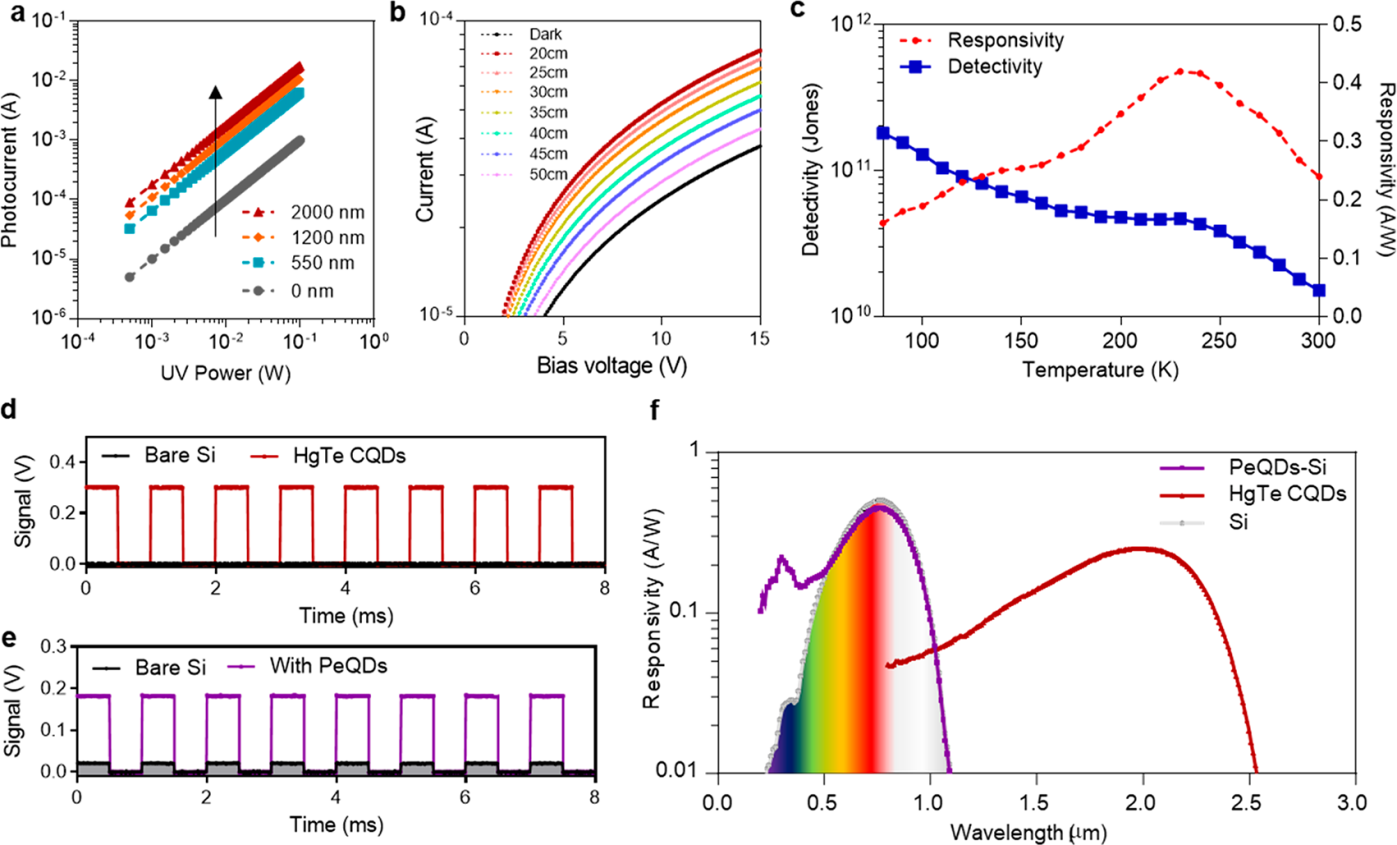
Characterization of Si, PeQD-Si, and HgTe CQD photodetectors. (a) Photocurrents as a function of UV intensity. (b) Measured *I*–*V* curves of HgTe CQD photoconductors. (c) Responsivity and detectivity as a function of operating temperature. (d) Measured response of a bare silicon detector and HgTe CQD-Si detector to chopped SWIR. (e) Measured response of a bare silicon detector and PeQD-Si detector to chopped UV light. (f) Responsivity as a function of wavelength.

Besides patterning resolution, the stability of optical and electronic properties of CQDs during UV patterning are also important figures of merit to be considered. One key prerequisite for sensitive UV detection is the high efficiency of PeQDs’ photoluminescence (PL). PeQDs have been reported with a high PL efficiency of up to 90%.^21,22^ After the addition of UV-activated ligands, the PL of PeQDs did not show any shift of emission wavelength or degradation of PL intensity, which is consistent with the PL lifetime measurements (Figure S4). Using a UV lamp with a wavelength of 375 nm as a light source, the photocurrents as a function of UV intensity of silicon photodiodes without PeQDs and with PeQDs with various thicknesses were measured (Figure. 2a). The results show that an over 1 order of magnitude improvement in photoresponse sensitivity from 0.01 to 0.18 A/W has been achieved. Compared with a bare imager without PeQDs, a significant increase of photocurrents can be observed after the addition of the PeQD film with a thickness of 550 nm. It is true that a further increased thickness of the PeQD film still leads to increases in the magnitude of photocurrent. However, a thicker PeQD film might be beneficial when the imager works with UV light with high intensity, which could lead to a higher saturation threshold. However, if the PeQDs film is too thick, the diffraction might cause optical crosstalk. Therefore, in our study, the film thickness of PeQDs was selected to be 1200 nm.

HgTe CQD photoconductors were fabricated by layer-by-layer UV exposure, followed by solid-phase ligand exchange with ethanediol in isopropanol. Field-effect transistor measurements show that the carrier mobility of HgTe CQDs with and without UV lithography is almost the same. The HgTe CQDs with UV-activated ligands remain photoconductive. The *I–V* curve of HgTe CQDs photoconductors illuminated with a blackbody (*T* = 600 °C) at a different distance were measured, as shown in Figure 2b. The responsivity of a HgTe CQD detector can be calculated by dividing its photocurrents by the incident optical power. The detectivity can be then calculated by
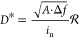
1where *A* is the area of the detector, Δ*f* is the bandwidth, and *i*_n_ is the noise. The responsivity and detectivity as a function of operating temperature are shown in Figure 2c. The bias voltage is set to be 3 V to be consistent with the maximum bias voltage of the ROICs. At room temperature, the responsivity and detectivity are as high as 0.24 A/W and 1.5 × 10^10^ jones. Within the reach of thermoelectric cooling down to 230 K, the responsivity and detectivity increase to 0.4 A/W and 5 × 10^10^ jones.

Figure 2d,e shows the response of bare silicon, HgTe CQD, and PeQD-Si detectors to chopped SWIR and UV light. It can be seen that the PeQDs can improve the photoresponse of Si photodiodes and that HgTe CQDs demonstrated much wider spectral response ranges than Si. Therefore, by combining PeQDs and HgTe CQDs, the sensing ranges of Si imagers can be greatly enhanced into the high-photon-energy and low-photon-energy ranges (Figure. 2f).

## 3 Single-Color Imagers

We then proceed to fabricate and characterize the single-color CQDs imagers. Unlike bulk semiconductors, CQDs can be processed by solution-phase methods. The array size of the imager used in our study is 320 × 256, with a pixel pitch of 30 μm. Details of the modification process of ROICs can be found in section S3 in the Supporting Information. A PeQD-ROIC imager was first fabricated. Uniform and crack-free PeQD films can be made. The thickness of PeQDs is ∼1200 nm.

**Figure 3:**
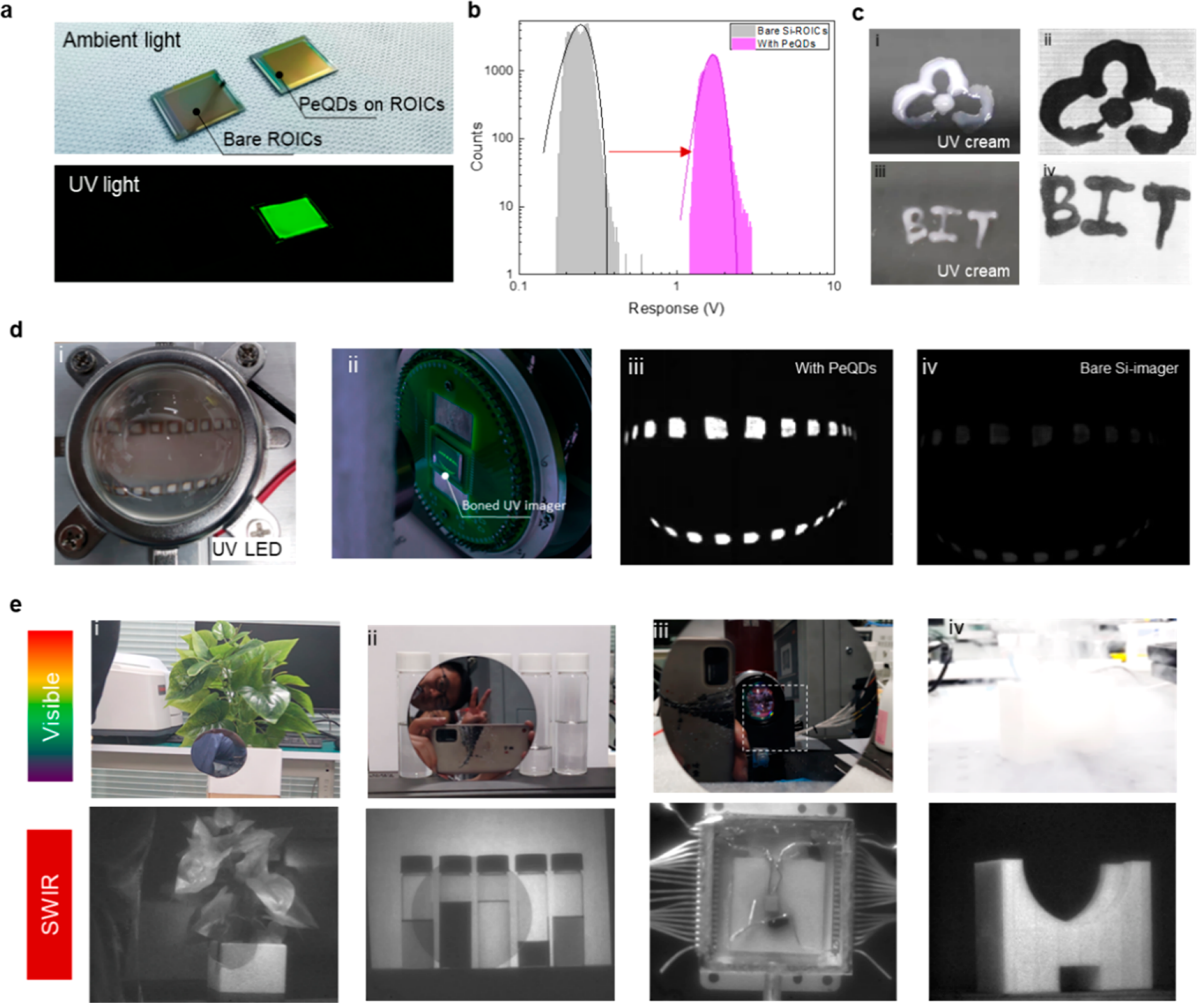
Single-color CQDs imagers. (a) ROIC chip images under ambient light and UV illumination. (b) Distribution of the response from bare ROICs and PeQD-ROICs. (c) Images of hand-drawn patterns of UV creams. (d) Visible and UV images of (i) UV LED imager, (ii) boned UV imager, (iii) PeQD-ROIC imager, and (iv) bare Si imager. (e) Visible and SWIR images by an HgTe CQD imager.

Figure. 3a shows the Si-ROIC chip with and without PeQDs under ambient light and UV illumination. For an array-format imager, uniform response across the sensing area is a prerequisite for imaging operation. With xenon lamp or blackbody as a light source, the response mapping of the UV and SWIR can be measured. The responsivity of a pixel can be calculated by
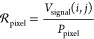
2where *V*_*signal*_(*i*,*j*) is the signal voltage of the *i*th row and *j*th column and *P*_pixel_ is the optical power incident on the pixel. The average responsivity of the imager is then calculated by
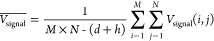
3where *M* is the total number of pixels in a row, *N* is the total number of pixels in a column, *d* is the number of dead pixels, and *h* is the number of overheated pixels. A dead pixel is defined as a pixel with a signal lower than 50% of the average signal. An overheated pixel is a pixel with noise 2 times higher than the average noise. The photoresponse nonuniformity (PRNU) can be calculated by
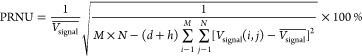
4

Figure 3b shows the summary of response signals of the silicon channels with and without PeQDs. The average response was improved from 0.25 to 1.86 V. After the addition of PeQDs, the PRNU was ∼5%, and one striking feature was that no dead pixel iwass found across the sensing area, benefiting from the uniform coverage of PeQDs on ROICs. As a visual demonstration of the imaging capability, images of hand-drawn patterns of UV creams were captured by using a UV lamp at 375 nm in reflectance mode (Figure 3c). The results show strong attenuation of the signal by the UV cream, confirming that the spectral sensing range is UV. We further compared the UV sensitivity between imagers with and without PeQDs. As shown in Figure 3d, an array of UV LEDs was imaged. Strong downconverted green PL can be observed from the imagers coated with PeQDs, which leads to a much-improved signal intensity and image contrast.

For SWIR imaging, external lighting from a tungsten lamp or sunlight was required, and all SWIR images were captured at room temperature. The SWIR imagers were made with UV-cured HgTe CQDs. As shown in Figure 3e, high-quality SWIR images were obtained. Compared to visible light, the SWIR images can see through opaque silicon, and transparent chemicals (left to right: chlorobenzene, ethanol, chloroform, water, butyl acetate) show different gray scales due to their variation in absorption strength. Benefiting from the longer wavelength, SWIR images demonstrated better penetration and image quality through fog.

To further improve the photoreponse of the multispectral imager, integration with optical structures could be a possible way to boost sensitivity by increasing the light absorption. Optical manipulation with plasmonic metallic structures,^23,24^ photonic crystals,^25,26^ metasurfaces, and immersion lenses have been widely used in photodetectors to boost device performance by increasing light absorption. Usually, the responsivity and quantum efficiency can be improved by near-field enhancement or light concentration. In our previous studies, resonant cavities have been integrated with HgTe CQD detectors and demonstrated over 200% enhancement in responsivity.^15,27^

## 4 Multispectral Imagers

**Figure 4:**
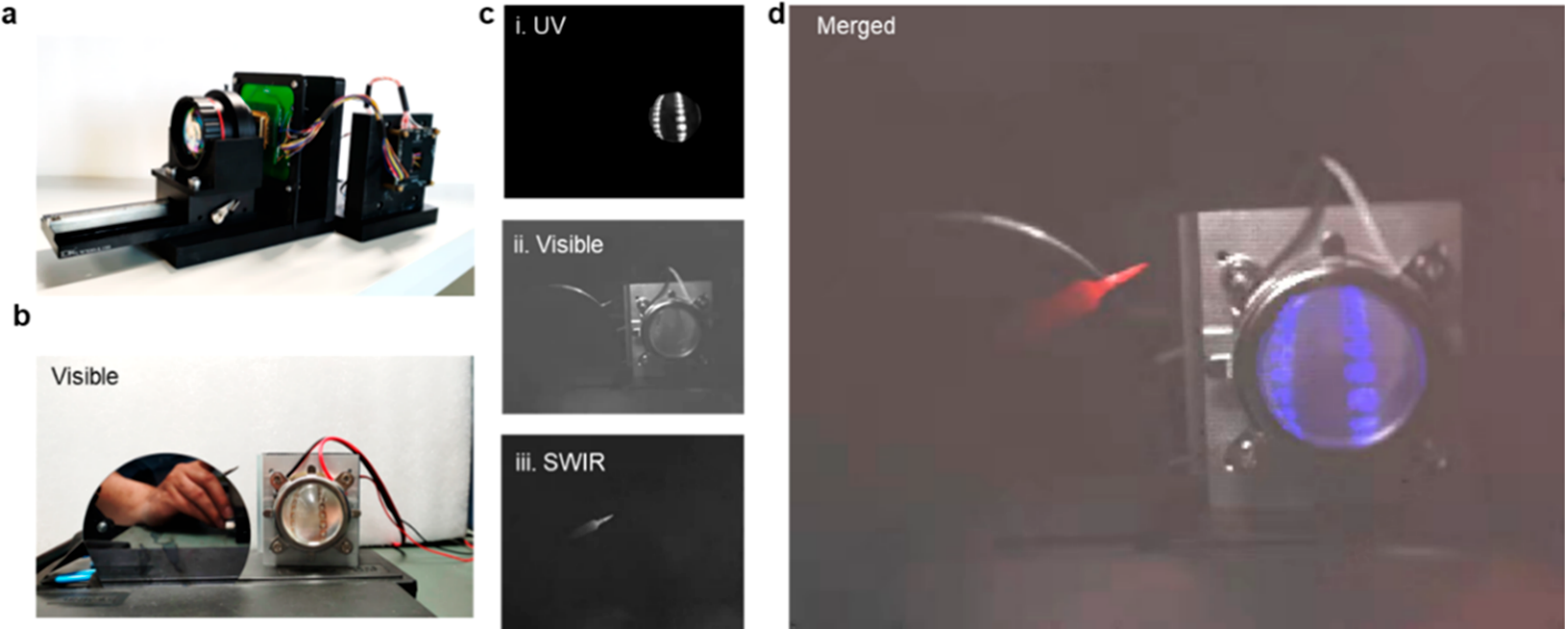
Multispectral colloidal quantum-dot imagers. (a) Multispectral colloidal quantum-dot imagers and periphery circuits. (b) Imaging scene: a soldering iron, a silicon wafer, and a UV lamp. (c) Captured UV, visible, and SWIR images. (d) Merged multispectral images with a UV image as the blue channel, visible light as the grayscale channel, and an SWIR image as the red channel.

Finally, we made a multispectral imager by patterning PeQDs and HgTe CQDs onto the same ROICs. Details of the fabrication process and fabricated imagers can be found in Figures S6 and S7. The effective image resolution for each channel is 160 × 256. It is worth noting that the resolution and pixel size of the CQD imagers are defined by the ROICs. Therefore, extending the image format and shrinking the pixel size should be easily realized with redesigned ROICs. An image demultiplexing periphery circuit was designed and could be used to output the raw and reconstructed images (Figure 4a). The imaging scene included a soldering iron with a temperature of 580 °C, a silicon wafer, and a UV lamp (Figure 4b). With a UV lamp on and ambient light off, a single-color UV image can be captured. Visible light can still be detected by the imager benefiting from the high transmittance of thin PeQDs. Figure 4c shows the single-color UV, visible, and SWIR images, highlighting the multispectral optical features. By using a UV image as the blue channel, visible light as the grayscale channel, and an SWIR image as the red channel, multispectral images can be reconstructed (Figure 4d). More importantly, the detectors show fast a response, and the measured response time is around 1 μs for HgTe CQD detectors, as shown in Figure S8.

Multispectral images that exceed single-color images can provide more details and avoid the complexity of the optical hardware and software, reducing the volume and weight of the multiband camera. Although two-color and three-color infrared focal plane arrays have been demonstrated,^28−30^ the combination of infrared bulk materials with UV sensing materials is inherently prohibited due to the mismatch of lattice constants. The direct optical lithography of CQDs enables the possibility of the manipulation of semiconductor sensing materials in a CMOS-compatible way.

## 5 Conclusions

In conclusion, we developed multispectral colloidal quantum-dot imagers by direct optical lithography. A multispectral colloidal quantum-dot imager constructed by patterning PeQDs and HgTe CQDs onto an Si imager extended the sensing ranges of Si-CMOS to the ultraviolet and SWIR (1.1–2.5 μm) and enabled facile discrimination of UV light, visible light, and SWIR. The multispectral colloidal quantum-dot imager exhibits high responsivity at the UV and SWIR (cutoff wavelength 2.5 μm). CMOS-compatible HgTe CQD and PeQD imagers avoid the complicated flip-bonding process, increase production yields, and reduce fabrication costs. Combining downconversion perovskite quantum dots and infrared colloidal quantum dots on the same Si-ROICs is a simple and successful method that provides a feasible approach for developing next-generation high-resolution, uncooled, low-cost multispectral imagers from the ultraviolet to short-wave infrared.

## 6 Materials and Methods

### 6.1 Synthesis of UV-Activated Ligands

The synthesis of UV-activated ligands of ethane-1,2-diyl bis(4-azido-2,3,5,6-tetrafluorobenzoate) was done following a modified method.^31,32^ The synthesis starts by dissolving 4-azido-2,3,5,6-tetrafluorobenzoic acid (940 mg) and thionyl chloride (1 M in dichloromethane) (25 mL) in anhydrous dichloromethane (32 mL) at 80 °C overnight. The reaction mixture was cooled to room temperature, and then the organic solvents were removed by distillation at reduced pressure. The resulting compound was dissolved in anhydrous dichloromethane (12 mL) and transferred to a mixture of ethylene glycol (103 mg) and triethylamine (402 mg) in anhydrous dichloromethane (18 mL). After it was stirred for 12 h at room temperature, the mixture was quenched by the addition of 1 M HCl(aq) (25 mL). The resulting solution was extracted three times with dichloromethane (16 mL). The dichloromethane phases were washed with brine (60 mL) and dried over anhydrous MgSO_4_. After filtration, the organic solvent was removed from the filtrate using a rotary evaporator at reduced pressure. The products were stored in a glovebox at temperatures below 2 °C.

### 6.2 Synthesis of Perovskite Colloidal Quantum Dots and HgTe CQDs

The CsPbX_3_ perovskite QDs were synthesized by a modified hot-injection procedure. In a typical experiment, PbX_2_ (0.36 mmol each for X = Cl, Br, or I), oleic acid (1.0 mL), oleylamine (1.0 mL), and octadecene (10 mL) were placed in a reaction bottle (50 mL) in a glovebox under a nitrogen atmosphere. The resulting mixture was heated to 100 °C with vigorous stirring for 0.5 h. Then the mixture was heated to 160 °C until the PbX_2_ precursors dissolved completely. Separately, Cs-oleate as a cesium precursor was prepared by placing Cs_2_CO_3_ (0.4 g, 1.23 mmol), oleic acid (1.25 mL), and octadecene (15 mL) in a reaction bottle in the glovebox. The reaction mixture was heated to 150 °C until the solution became clear. The Cs-precursor solution was kept at 150 °C under a nitrogen atmosphere. To initiate the reaction, a hot Cs-oleate precursor solution (1 mL) was injected quickly into the PbX_2_ precursors. After 5 s of reaction, the flask was transferred into an ice bath. The CsPbX_3_ QDs were obtained by precipitation and centrifugation and stored in 4 mL of hexane before further use. For HgTe CQDs, HgCl_2_ (0.15 mmol) was dissolved in 4 g of oleylamine in a 20 mL glass vial at 100 °C for 30 min with stirring in the glovebox. Tellurium in trioctylphosphine (TOP) solution (1 M, 0.15 mL) was rapidly injected after adjusting the temperature of the reaction mixture to 80 °C. The clear solution immediately turned black. The reaction was quenched by injecting a solution of 0.4 mL of dodecanethiol (DDT) and 0.16 mL of TOP in 4 mL of tetrachloroethylene (TCE). Around 2 mL of a crude solution of HgTe CQDs was diluted with 2 mL of TCE, 0.3 mL of TOP, and 0.3 mL of DDT. The solution was precipitated with an equal volume of isopropanol (IPA) and centrifuged at 4500 rpm for 2 min before it was resuspended in 4 mL of chlorobenzene. Before optical lithography, the UV-activated ligands were added to the CQD solution (2 wt %).

### 6.3 Fabrication and Characterization of Single-Element Detectors

The PeQD-enhanced silicon photodiodes were fabricated by spin-coating a PeQD solution (200 mg/mL) with UV-activated ligands followed by UV exposure to cure the films. To build PeQD films with thicknesses up to 1000–2000 nm, three to five layers were added. The HgTe CQD detectors were fabricated by spin-coating HgTe CQDs onto a sapphire chip with predefined interdigitated electrodes. The concentration was ∼100 mg/mL, and the HgTe CQDs were spin-coated at 1000 rpm. Four layers of HgTe CQDs were added. Each layer was cured by UV exposure followed by solid-phase ligand exchange with ethanedithiol (EDT)/HCl/IPA (1/1/50 by volume) solution for 10 s. In the last step, n-type HgTe CQDs were added to make a trapping-mode photoconductive CQD detectors.^33^ The n-type HgTe CQDs were synthesized by a previously reported procedure.^34^ To measure the photoresponse, blackbody and xenon lamps were used as the infrared and UV light sources, respectively. The photocurrent was amplified first by a preamplifier and then by a voltage amplifier and was received by an oscilloscope.

### 6.4 Fabrication and Characterization of Single-Color and Multispectral Imagers

The single-color imagers were fabricated by the same procedure as single-element detectors. For the multispectral imagers, the HgTe CQD pixels were first patterned by repeating the alignment, UV exposure, and ligand exchange processes. In the lithography process, chlorobenzene was used as the developer to remove the unexposed area of CQDs. The PeQD pixels were then added by spin-coating, UV exposure, and development. The imaging of CQD imagers was conducted with a focal plane array tester with an uncoated quartz lens, which can provide power, ground, timing, and trigger signal. The output channels from the ROICs were sampled and reordered to construct raw images and merged images. The performance, at both the array and pixel levels, can be assessed. The RMS noise, fixed pattern noise, crosstalk, pixel surface response, and detectivity can be generated.

## Supporting Information Available

Characterization of UV-activated ligands, Ccharacterization of PeQDs with and without UV-activated ligands, modification of silicon readout integrated circuits (ROICs), fabrication process of perovskite CQD and HgTe CQD pixels, characterization of multispectral imagers, and response speed measurement (PDF)
