# Supplementary material for: Direct Optical Lithography Enabled Multispectral Colloidal Quantum-Dot Imagers from Ultraviolet to Short-Wave Infrared
Source: ACS Nano. 2022 Nov 8;16(11):18822–9. doi: 10.1021/acsnano.2c07586 (PMC9706660; doi:10.1021/acsnano.2c07586)
Supplement: Supplementary file 1 — nn2c07586_si_001.pdf [file nn2c07586_si_001.pdf]

Direct Optical Lithography Enabled Multispectral Colloidal Quantum-dot Imagers from Ultraviolet to Short-wave infrared

*Shuo Zhang[1,#], Cheng Bi [1,2,#], Yimei Tan[1], Yuning Luo[1], Yanfei Liu[2], Jie Cao[1,3,4], Menglu Chen[1,3,4*], Qun Hao[1,3,4*], Xin Tang[1,3,4*]*

[1] School of Optics and Photonics, Beijing Institute of Technology, Beijing, 100081, China.

[2] Zhongxinrecheng Science and Technology co.,ltd, Beijing, 101102, China.

[3] Beijing Key Laboratory for Precision Optoelectronic Measurement Instrument and Technology, Beijing, 100081, China.

[4] Yangtze Delta Region Academy of Beijing Institute of Technology, 314019, China.

**S1. Characterization of UV-activated ligands**

**S2. Characterization of PeQDs with and without UV-activated ligands**

**S3. Modification of silicon readout integrated circuits (ROICs)**

**S4. Fabrication process of perovskite CQDs and HgTe CQDs pixels**

**S5. Characterization of multispectral imagers**

**S6. Response speed measurement**

## S1. Characterization of UV-activated ligands

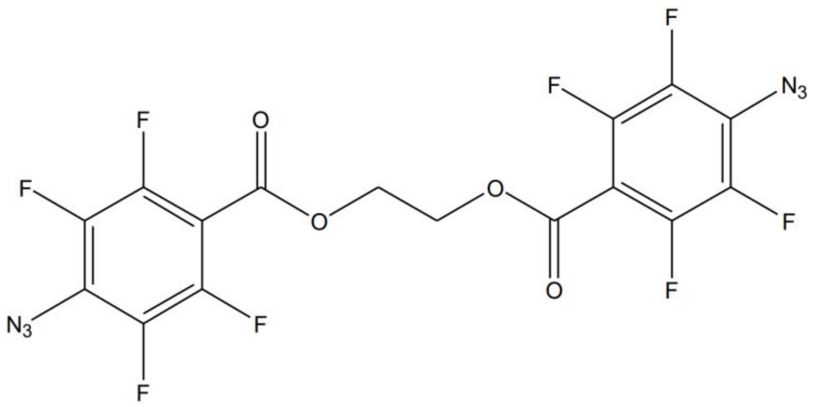


**Figure. S1** Chemical structure of UV-activated ligands.

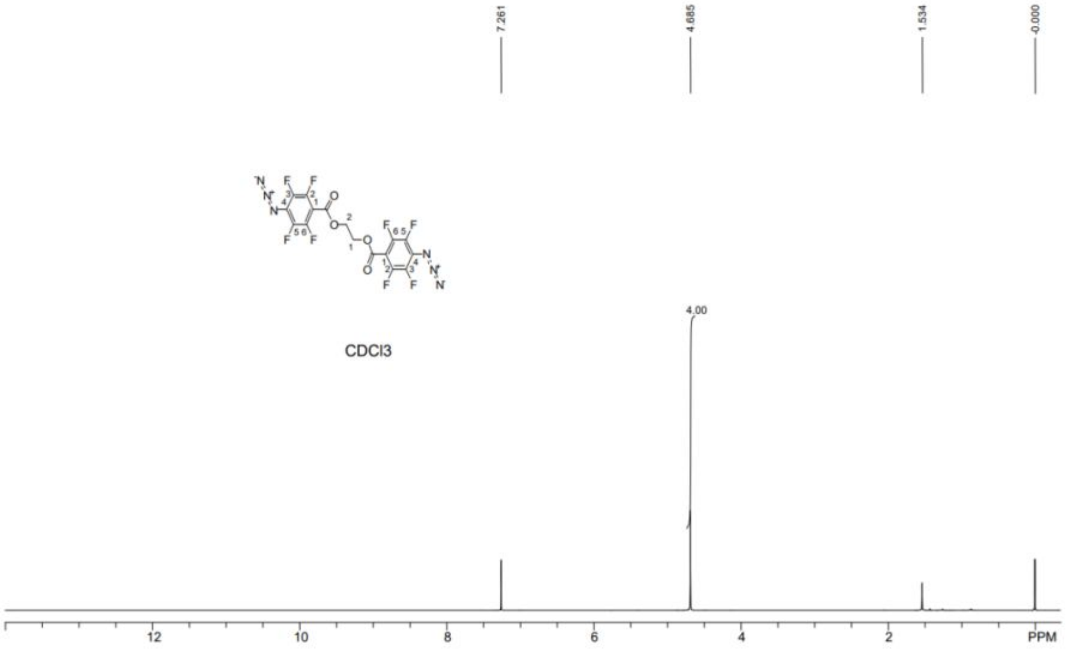


**Figure. S2**. The HNMR spectrum of the UV-activated ligands.

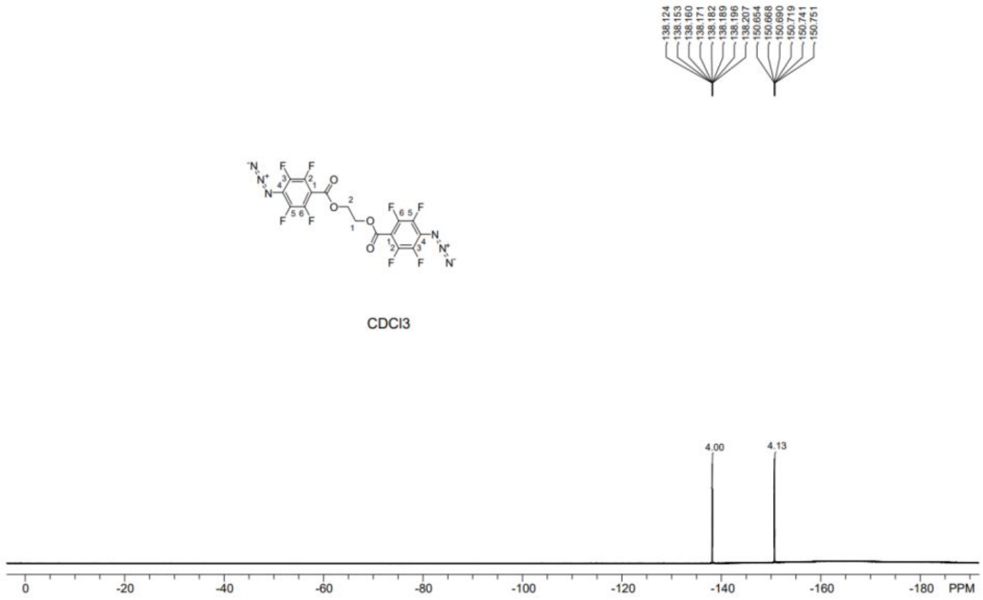


**Figure. S3** The FNMR spectrum of the UV-activated ligands.

**S2. Characterization of UV-activated ligands**

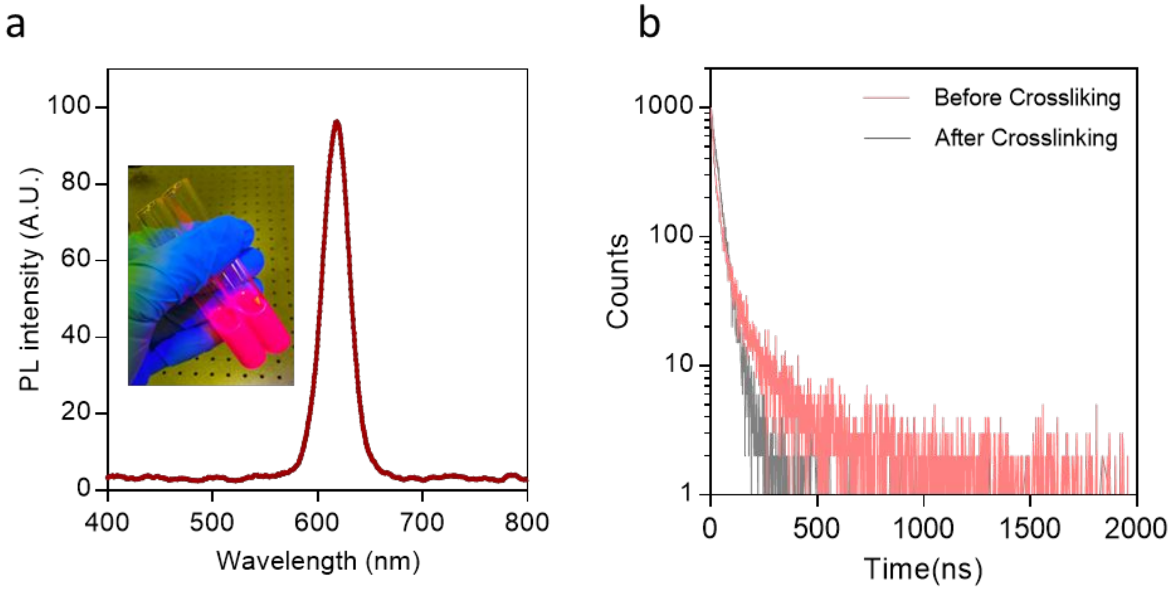


**Figure. S4** a. PL of PeQDs solution with and without UV-activated ligands. b. Lifetime of PeQDs with and without UV-activated ligands.

**S3. Modification of silicon readout integrated circuits (ROICs)**

The proposed multispectral imagers relied on a readout circuit with two types of pixels: a visible pixel to detector the photoluminescence from perovskite QDs and an amplification pixel to integrate photocarriers from HgTe CQDs and convert the charges to voltages. However, silicon readout integrated circuits (ROICs) with such hybrid configuration remain unavailable. Therefore, in our study, we modify a commercially available ROICs to realize such functionalities. The ROICs contains 320×256 amplification pixels with direct injection mode. An integral capacitor is fabricated in each pixel. For a photodiode, it needs two electrodes to connect with the ROICs. The 320×256 of pixel electrodes are arranged in the center part of the ROICs, while the ground electrodes are arranged around the pixel electrode area as shown in the **Figure S5a**.

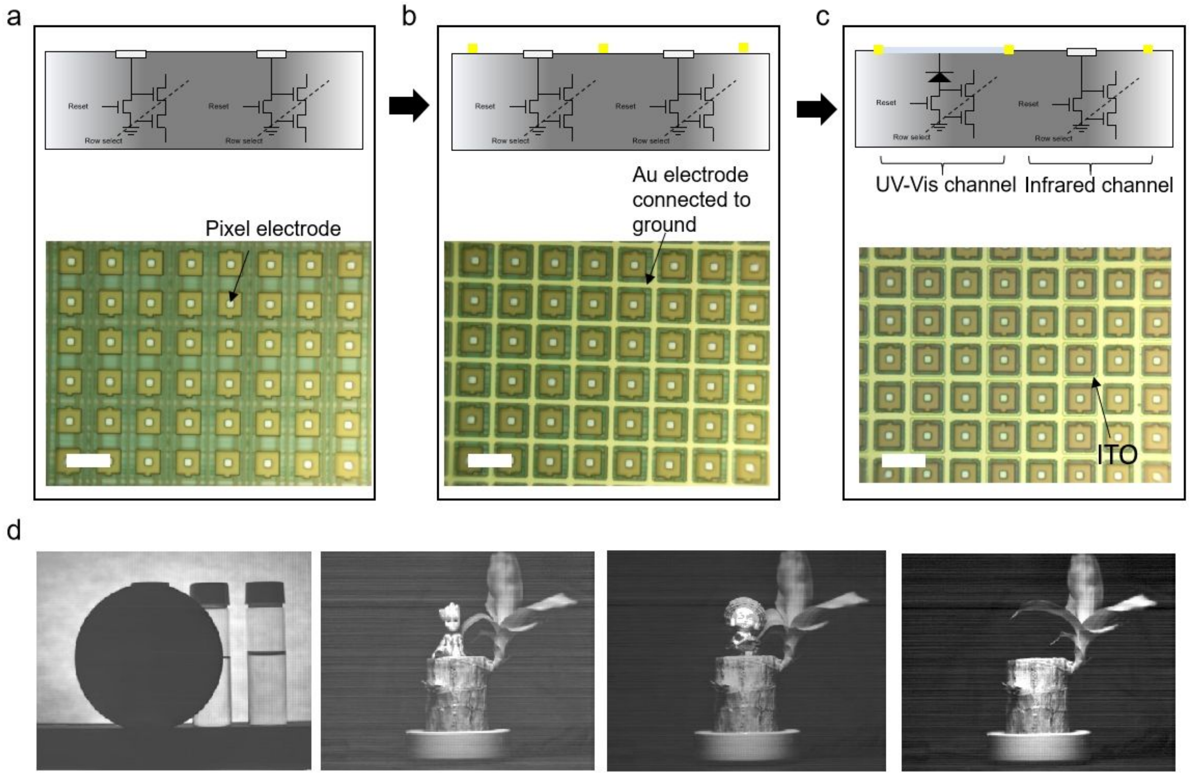


**Figure. S5** a. Bare ROICs with pixel electrode array. b. ROICs with pattered ground electrode. c. ROICs with ground electrode and deposited ITO contacts. The scale bar is 30μm. d. Captured visible images with a modified ROICs with ITO top contact.

To modify the ROICs to meet our requirement for the hybrid configuration with visible pixel and amplification pixels, the ground electrode is first patterned and deposited around each pixel electrodes, which eliminate any transportation of photocarriers between adjacent pixels (**Figure. S5b** ). Then, transparent and conductive indium Tin oxides (ITO) electrodes are deposited on the ROICs column by column (**Figure. S5c**). The ITO short the ground and pixel electrodes. Visible photons can pass the ITO contact and will be absorbed by the underlying silicon layers, diodes or field-effect transistors. The visible photons excite carriers, which charge the integral capacitors and form visible images. For the single color imager with provskite QDs, all the pixels are covered with ITO contacts. As shown in **Figure. S5d**, the modified ROICs can sense visible light and output visible images.

### S4. 2.Fabrication process of perovskite CQDs and HgTe CQDs pixels

The multispectral imagers consist of perovskite QDs pixels and HgTe CQDs pixels. Each layer of the deposited HgTe CQDs need to be treated with ethanedithiol (EDT)/HCl in isopropanol (2% volume ratio). As the EDT could significantly quench the photoluminescence of perovskite QDs, HgTe CQDs pixels are fabricated before the perovskite QDs pixels.

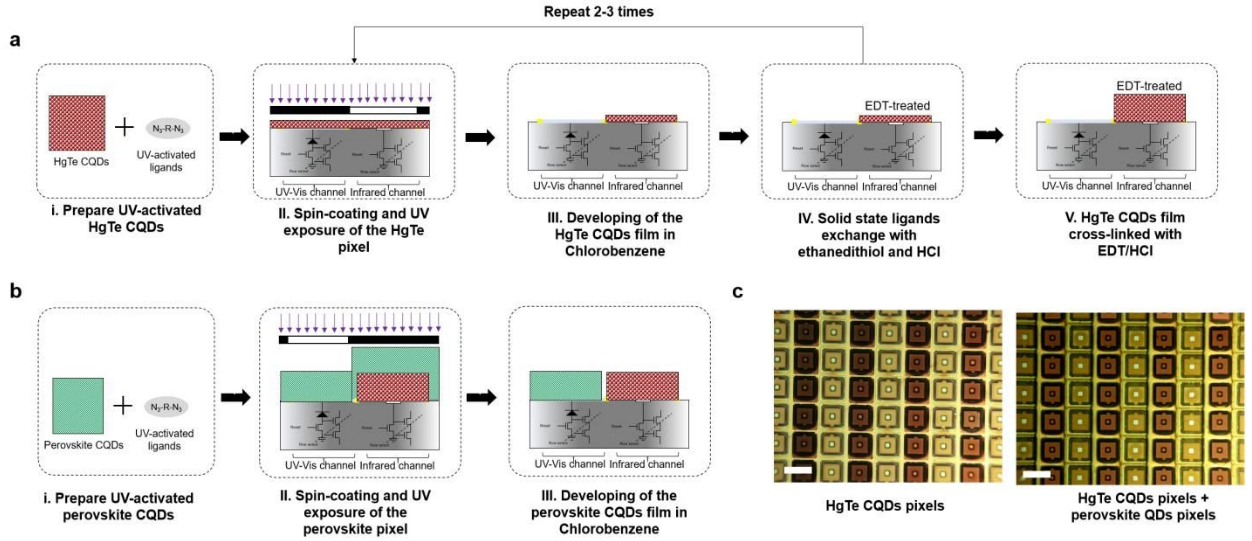


**Figure. S6** a. Fabrication process of HgTe CQDs pixels. b. Fabrication process of perovskite QDs pixels. c. Optical images of ROICs with HgTe CQDs pixels and perovskite QDs pixels. The scale bar is 30μm.

The fabrication process starts from the synthesis of UV-activated HgTe CQDs solution. The 2%wt UV ligands are dissolved in the HgTe CQDs solution. The UV-activated HgTe CQDs

solution is then spin-coated onto the modified ROICs, as shown in **Figure. S6a**. After that, the HgTe CQDs film is then exposed with UV light with dose of 200 mJ through a photomask. After exposure, the HgTe CQDs are developed with Chlorobenzene, resulting in patterned HgTe CQDs pixels. Then, the patterned HgTe CQDs film is then treated with EDT/HCl solution. After EDT/HCl treatment the HgTe CQDs become photoconductive. The spin-coating, UV exposure and developing process can be repeated multiple times until the HgTe CQDs films reach to 300-400 nm in thickness.

The perovskite QDs pixels are fabricated by the same processes of spin-coating, UV exposure, and developing, as shown in **Figure. S6b**. **Figure. S6c** shows the optical images of ROICs with HgTe CQDs pixels and ROICs with both HgTe CQDs pixels and perovskite QDs pixels.

### S5. Characterization of multispectral imagers

Optical microscope images of the multispectral imager under ambient light and UV light.

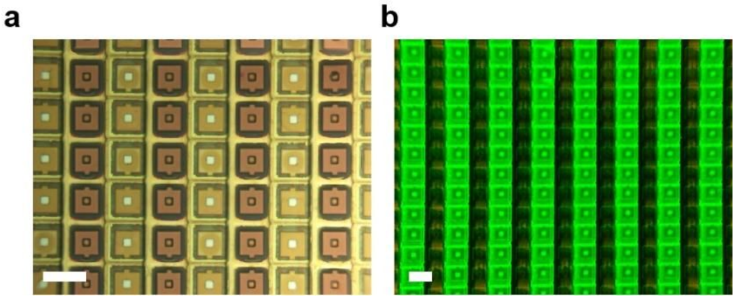


**Figure. S7**. a. Optical image of ROICs with HgTe pixels and perovskite QDs pixels under ambient light. b. Optical image of ROICs with HgTe pixels and perovskite QDs pixels under UV lightThe scale bar is 30μm.

### S6. Response speed measurement

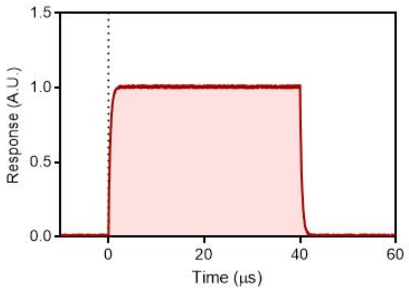


**Figure. S8**. response speed measurement.
